# Novel noncontact charge density map in the setting of post-atrial fibrillation atrial tachycardias: first experience with the Acutus SuperMap Algorithm

**DOI:** 10.1007/s10840-020-00808-9

**Published:** 2020-07-08

**Authors:** Robbert Ramak, Gian-Battista Chierchia, Gaetano Paparella, Cinzia Monaco, Vincenzo Miraglia, Federico Cecchini, Antonio Bisignani, Joerelle Mojica, Maysam Al Housari, Dimitrios Sofianos, Shuichiro Kazawa, Ingrid Overeinder, Gezim Bala, Erwin Ströker, Juan Sieira, Thiago Guimaraes Osorio, Pedro Brugada, Carlo de Asmundis

**Affiliations:** Heart Rhythm Management Center, Postgraduate program in Cardiac Electrophysiology and Pacing, European Reference Networks Guard-Heart, Universitair Ziekenhuis Brussel, Vrije Universiteit Brussel, Laarbeeklaan 101, 1090 Brussels, Belgium

**Keywords:** SuperMap algorithm, Dipole density noncontact mapping, Unipolar signals, Atrial tachycardia, Contact force ablation, LSI

## Abstract

**Purpose:**

The purpose of this study was to evaluate the safety and feasibility of the new high-resolution mapping algorithm SuperMap (Acutus Medical, CA, USA) in identifying and guiding ablation in the setting of regular atrial tachycardias following index atrial fibrillation (AF) ablation.

**Methods:**

Seven consecutive patients who underwent a radiofrequency catheter ablation guided by the novel noncontact charge density (CD) SuperMap for atrial tachycardia were prospectively enrolled in our study.

**Results:**

Arrhythmogenic substrate was identified in all seven patients. Mean number of EGM per map was 5859.7 ± 4348.5 points. Three patients (43%) exhibited focal tachycardia mechanisms in the left atrium, alternating from anteroseptal right superior pulmonary vein (RSPV), posterior in proximity of left inferior pulmonary vein (LIPV), and interarial septum in proximity of fossa ovalis, respectively. Four patients exhibited macroreentrant mechanism. In 3 of these patients, SuperMap detected mitral isthmus-dependent flutters with tachycardia cycle lengths of 240, 270 and 420 ms, respectively. In one patient, the mechanism was a macroreentrant tachycardia with the critical isthmus located between the crista terminalis and atriotomy. The mean ablation time (min) was 18.2 ± 12.5 and the mean procedural duration time was 56.4 ± 12.1 min. No minor or major complications occurred.

**Conclusion:**

The novel high-resolution mapping algorithm SuperMap proved to be safe, fast, and feasible in identifying and guiding ablation in the setting of regular atrial tachycardias following index AF ablation.

## Introduction

Atrial tachycardias (AT) can be observed following ablation of atrial fibrillation (AF) in 5–25% of cases [1–4]. The underlying mechanisms comprise focal AT, micro re-entry, and more often macro re-entrant circuits [5]. Although three-dimensional (3D) mapping is of great use in mapping AT, ablation of these remains arduous [6]. Mapping such irregular rhythms is technically challenging with the current sequential 3D mapping systems and often burdensome and time-consuming, especially in case of variations of the tachycardia cycle length [7–9]. The AcQMap 3D imaging and mapping system (Acutus Medical, CA, USA) utilizes the unique combination of ultrasound and non-contact global electrical mapping [10, 11]. The new mapping system uses ultrasound to reconstruct the atrial chamber anatomy to create a computed tomography (CT) quality like anatomy. An inverse solution uses the noncontact unipolar voltage electrodes to derive charge density (CD) propagation maps in order to visualize complex arrhythmias, without the confounding effects of far field signals [12]. A recent addition to the AcQMap System is the novel SuperMap algorithm. The latter has been created to study regular atrial rhythms. SuperMap is a recent addition that allows accurate and efficient treatment of both sustained and transient regular arrhythmias by the means of a unique noncontact “hover-map” approach. The simultaneous and global nature of noncontact mapping may help to understand the mechanisms responsible for initiating and maintaining these arrhythmias [13]. This might also lead to less time-consuming procedures. To the best of our knowledge, mapping and ablation of regular atrial tachycardias following atrial fibrillation ablation with the novel noncontact CD SuperMap algorithm have not been described yet in a series of consecutive patients.

## Aim of the study

The aim of the study is to evaluate the safety and feasibility of the new high-resolution mapping algorithm SuperMap in identifying and guiding ablation in the setting of regular atrial tachycardias following index AF ablation.

## Methods

### Study population

The study cohort consisted of consecutive patients presenting with AT post AF ablation with the cryoballoon (Artic Front Advance, Medtronic, MN, USA) who underwent a radiofrequency catheter ablation guided by the novel noncontact CD mapping technology at the Heart Rhythm Management Center, UZ Brussels, Belgium, between December 2019 and April 2020. The study was retrospective in nature and approved by the local ethics committee of our Institution.

### Exclusion criteria

The exclusion criteria were the following: nontreated coronary artery disease, intracavitary thrombus, and contraindications to general anesthesia.

### Pre-procedural management

All patients provided written informed consent to the ablation procedure. A transthoracic echocardiogram (TTE) was performed within 1 week prior to ablation enabling assessment of structural heart disease. All antiarrhythmic drugs (AADs) were discontinued at least 3 days before ablation. To exclude the presence of thrombi, transesophageal echocardiography (TEE) was performed the day before the procedure. If a recent CT scan (prior to the index AF ablation) was not available, patients underwent the latter to assess detailed right atrium (RA), left atrium (LA), and pulmonary vein (PV) anatomy.

### Post-procedural management

After the procedure, patients were continuously monitored via telemetry for at least 18 hours. Before discharge, a TTE was performed in all patients in order to exclude post-procedural complications. Patients were discharged on the following day and were instructed to continue AAD and anticoagulation therapy for at least 3 months. After discharge, patients were scheduled for follow-up visits with baseline electrocardiogram (ECG) and 24-h holter recordings at 3, 6, and 12 months.

## Procedure

### Index procedure cryoballoon ablation

Our standard pre-procedural management and ablation has been previously reported in detail [14, 15]. After having accessed the LA, a 70 UI/kg heparin intravenous bolus was given, and activated clotting time was maintained over 250 s during the whole procedure. After having obtained LA access, through a steerable 15 Fr sheath (FlexCath Advance, Medtronic MN, USA), an inner lumen mapping catheter (Achieve, Medtronic, MN, USA) was advanced in the LA and was positioned in each pulmonary vein (PV) ostium. A 28-mm cryoballoon (Arctic Front Advance, Medtronic, MN, USA) was advanced inflated and positioned in each PV ostium. Optimal vessel occlusion was defined by selective contrast injection showing total contrast retention with no backflow into the LA. The ablation sequence was: treating the left superior PV (LSPV) first, followed by the left inferior PV (LIPV), right inferior PV (RIPV), and right superior PV (RSPV). Once vessel occlusion was deemed satisfactory, delivery of cryoenergy to allow freezing was commenced. Standard cryothermal applications lasted 180 s. Our target temperature was −40 °C within the first 60 s. If the temperature did not attain this value, an extra freeze was delivered. Successful pulmonary vein isolation (PVI) was defined as an absence of all PV potentials or their dissociation from an atrial activity. In order to avoid phrenic nerve palsy (PNP), diaphragmatic stimulation was achieved by pacing the phrenic nerve during septal PVs ablation.

## Repeat procedure

### Electroanatomical mapping via AcQMap

**Fig. 1:**
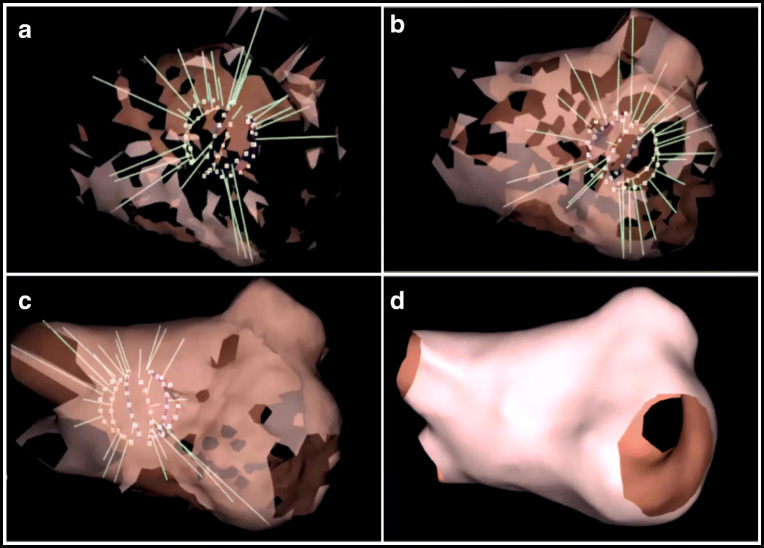
Anatomy reconstruction panels (**a**), (**b**), and (**c**) represent the build out of the LA anatomy with the ultrasound enabled AcQMap catheter. The green vectors on the map are originating from the 48 ultrasound transducers on the AcQMap catheter. The vectors are proportional to the distances travelled from the transducers towards the endocardial surface. Panel (**d**) represents the completed LA anatomy which will serve to project upon the electrical activation of the LA

Prior to mapping a 6F quadripolar catheter (MultiCath 6F, Biotronik, Berlin, Germany) was placed in the inferior vena cava below the level of the diaphragm with 1 electrode used as a universal electrical reference. A 6F decapolar catheter (ViaCath NG, Biotronik, Berlin, Germany) was positioned in the coronary sinus (CS) through the left femoral vein for CS reference. Prior to LA mapping, a circular mapping catheter (Reflexion Spiral, Abbott, MN, USA) was positioned in each vein to assess PVI, prior to mapping and ablation of the AT. For LA mapping, a transseptal puncture was performed under TEE guidance, with insertion of the AcQMap mapping catheter (AcQMap; Acutus Medical, CA, USA) via a customized steerable sheath (AcQGuide; Acutus Medical, CA, USA). The AcQMap catheter was positioned in the atrial chambers of interest to generate 3D surface reconstructions and maps of atrial activation. The AcQMap 3D Imaging and Mapping System uses the AcQMap catheter to reconstruct the endocardial anatomical surface and then overlays with high-resolution CD maps of electrical activation. By moving and rotating the AcQMap catheter, the 48 ultrasound transducers are used to reconstruct the atrial chamber anatomy (Fig. 1). Acoustic waves travel from the ultrasound transducers towards the myocardial surface. A portion of the wave continues to travel across the cardiac tissue, while a portion is reflected by the myocardial wall. The duration of time for the acoustic wave to travel from the transducer to the myocardial surface and return to the transducer is proportional to the distance travelled. The ultrasound waves/pulses continuously reach and reflect the chamber walls and accumulate up to 144,000 points every minute. The 48-engineered bio-potential electrodes record unipolar intracardiac voltage potentials to calculate cardiac activation as CD via the inverse solution. Electrical activity can be displayed as CD- or voltage-based maps. The activation wave front can be displayed in its rawest form as either a charge- or voltage-based map of depolarization. A propagation history map uses bands of color to show the location and velocity of the leading edge of the activation wave front over a set duration of time. The color red is used to indicate the leading edge of the wave front with the trailing color bands showing earlier locations of the wave front. The width of the color bands conveys the conduction velocity of the wave front, with wider bands indicative of fast conduction and narrow bands of slow conduction.

### SuperMap

**Fig. 2:**
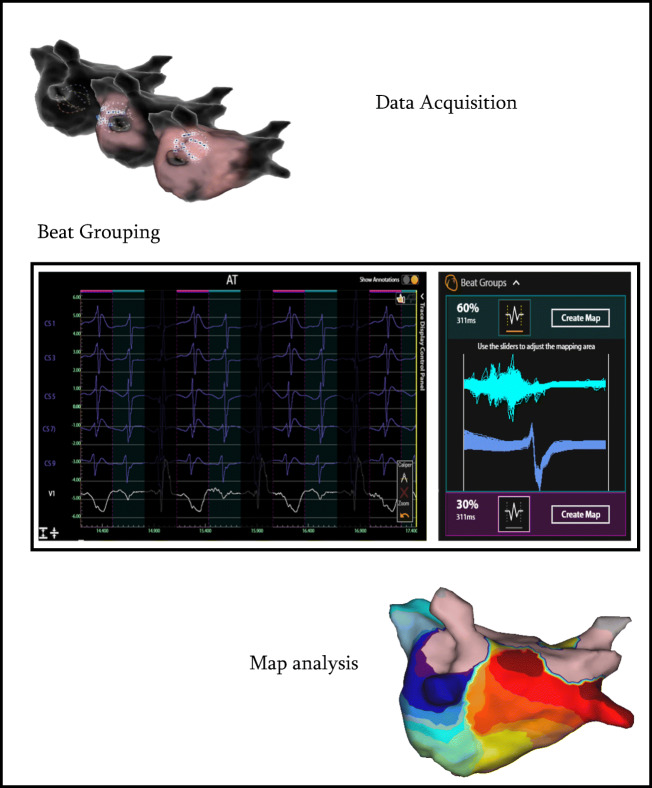
Overview workflow SuperMap. Step 1: AcQMap catheter hovering across the chamber of interest based on visual data acquisition workflow. Step 2: Automatic Beat Grouping based on uni- and bipolar reference signals that share similar morphology. Step 3: Automatic map creation associated with different beat groups are visualized for conduction wave front analysis

SuperMap is a novel algorithm within the AcQMap system designed to enable mapping of stable or transient rhythms with multiple morphologies. After hovering around the AcQMap catheter in the chamber of interest, multiple noncontact catheter positions are time aligned based on CS activation. Bi- and unipolar electrograms from CS were analyzed based on morphology into beat groups. Selected beat groups were processed through the CD inverse solution and visualized for conduction wave front analysis (Fig. 2).

## Site of ablation

### Re-entrant tachycardia

After the completion of the SuperMap, entrainment mapping on the visually localized critical isthmus was performed from the distal ablation bipole (TactiCath SE, Abbott, MN, USA) in order to confirm the findings. Entrainment was performed at multiple candidate sites at a pacing cycle length 15–20 ms shorter than TCL and high output (25 mA) [16]. Postpacing intervals (PPIs) were measured as the time (ms) between the last pacing stimulus and the first non-entrained electrogram deflection on the pacing electrode (return cycle). Ablation was performed on the critical isthmus.

### Focal tachycardia

Once the completion of the SuperMap, the site of ablation was tagged on the Acutus CD map which corresponded to the earliest activation site preceding the *P* wave onset by at least 30 ms and with a unipolar electrogram demonstrating a QS configuration with a steep negative deflection preceding the *P* wave.

### Ablation

A contact force (CF) catheter (TactiCath SE Abbott MN, USA) was used in all cases, and ablation was guided by lesion size index (LSI). In the LA the LSI target was 4.5 in the posterior wall and 5 on the anterior wall, roof, and floor. No LSI algorithm is currently validated for the RA [17–19]. That caused RA ablation to be guided by classic parameters: impedance drop and local voltage potential abatement.

In this case, the CF catheter was visualized and directed in the area of interest where double and fragmentated potentials were recorded on the AcQMap. Radio frequency (RF) energy was delivered in a power control mode and was limited to 35 watt (W). Irrigated flow ranged between 2 mL/min (standby), 17 mL/min (< 30 W), and 30 mL/min (> 30 W).

### Induction protocol following ablation

Pharmacological stimulation was performed with isoproterenol that was infused at 5, 10, 15, and 20 mcg/min at 2-min intervals or until AF was induced or a decrease in systolic blood pressure to < 85 mmHg was noted.

## Study endpoint

The endpoint of ablation was the conversion of AT to sinus rhythm and no arrhythmia inducibility following resumption to sinus rhythm.

## Complications

Those that were life-threatening, caused permanent harm, required intervention, or prolonged hospitalization such as pericardial effusion requiring percutaneous or surgical drainage or repair; peripheral vascular complication due to groin puncture requiring blood transfusion or arteriovenous fistula/pseudoaneurysm treated with percutaneous or surgical intervention or longer hospital stay; any thromboembolic event including transient ischemic attack (TIA) or stroke; atrioesophageal fistula; retroperitoneal hematoma; pleural hematoma; and death were considered to be major complications. Those that caused no permanent harm and were reversible such as local hematoma in the puncture site and very slight post-ablation pericardial effusion not requiring any intervention were considered minor complications.

## Results

### Baseline characteristics

A total of 7 patients, (mean age 67.6 ± 7.6; 85.7% males) were included. All had undergone a previous PVI with CB technology for paroxysmal AF (PAF). All patients had previously failed ≥ 1 Class I or III AAD trial following index procedure. Two patients exhibited prior history of hybrid thoracoscopic-transcatheter ablation for persistent atrial fibrillation. Mean time from index ablation to repeat ablation was 25.8 ± 14.4 months. The baseline and procedure characteristics are shown in Table 1.

**Table 1:** Baseline characteristics of the study population

Age (years)	67.6 ± 7.6
Gender (male)	6 (85.7%)
Body mass index, kg/m2	25.5 ± 2.2
Hypertension (%)	4 (57.1%)
Diabetes (%)	2 (28.6%)
Dyslipidemia (%)	4 (57.1%)
Antiarrhythmic drugs (%)	7 (100%)
Left ventricular ejection fraction (%)	52.9% ± 4.7

### Main procedural characteristics

All 28 pulmonary veins where permanently isolated at the time of the repeat procedure. Mean time for anatomy acquisition(s) with the AcQMap system was 166.0 ± 28.9 s. Mean mapping time (s) for each AT with the SuperMap algorithm was 326.6 ± 54.8 s. The mean total mapping time was 492.6 ± 57.7 s. The mean number of EGM points acquired by SuperMap was 5859.7 ± 4348.5 points. The mean total procedural duration time was 56.4 ± 12.1 min. Mean fluoroscopy time was 13.6 ± 9.49 min. The procedure characteristics are shown in Table 2.

**Table 2:** Main procedural characteristics of the study population

Mean Anatomy acquisition (s)	166.0 ± 26.9
Mean Mapping time (s)	326.6 ± 54.8
Mean total mapping time (m)	8.02 ± 1.0
Mean EGM points	5859.7 ± 4348.5
Mean Fluoroscopy time (min)	13.6 ± 9.4
Mean application/ablation points	30.0 ± 25.0
Mean ablation time (min)	18.2 ± 12.5
Mean procedural duration time (min)	56.4 ± 12.1
Time to Redo (months)	25.8 ± 14.4

### Tachycardia characteristics and mapping

**Fig. 3:**
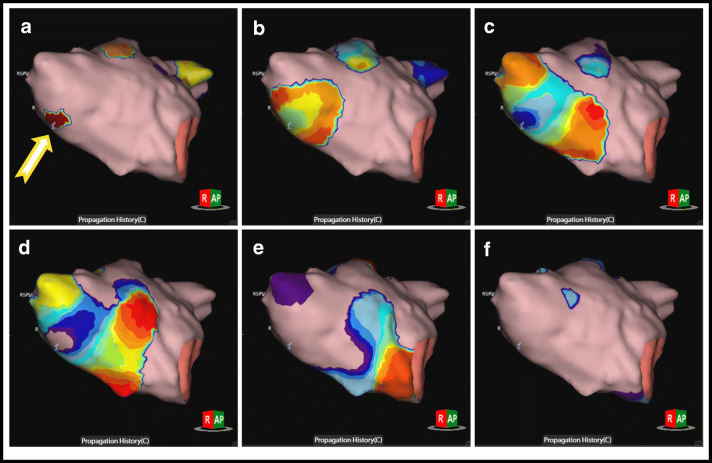
Focal AT: Panels (**a**), (**b**), (**c**), (**d**), (**e**), and (**f**) represent the electrical activation projected on the LA anatomy in different time steps throughout the full CL of the focal AT origination form underneath the RSPV (yellow arrow is starting point). All panels show a RAO view of the LA

The SuperMap algorithm successfully identified the arrhythmogenic substrate in all 7 patients (100%). Three patients (43%) exhibited focal tachycardia mechanisms. One patient presented with a focal tachycardia with the earliest activation in the anteroseptal region of RSPV with a cycle length (CL) of 270 ms (Fig. 3). Ablation in the earliest site (−35 msec before *P* wave) led to immediate termination of the arrhythmia. Isoproterenol infusion led to the induction of a second focal tachycardia located in the anterior wall of the LA (CL = 320 ms). Ablation in the earliest site led to conversion to sinus rhythm. Pharmacological stimulation could not induce further arrhythmias in this patient. In another patient, a focal tachycardia at a CL of 400 ms was localized on the posterior wall in proximity of the LIPV. Ablation led to conversion to sinus rhythm. No further arrhythmia was induced following ablation. The last patient presented with a focal tachycardia originating from the interatrial septum in proximity of the fossa ovalis at a mean CL of 235 ms. Applications on the left side of interatrial septum in this location led to the interruption of the arrhythmia. No further arrhythmias were induced.

**Fig. 4:**
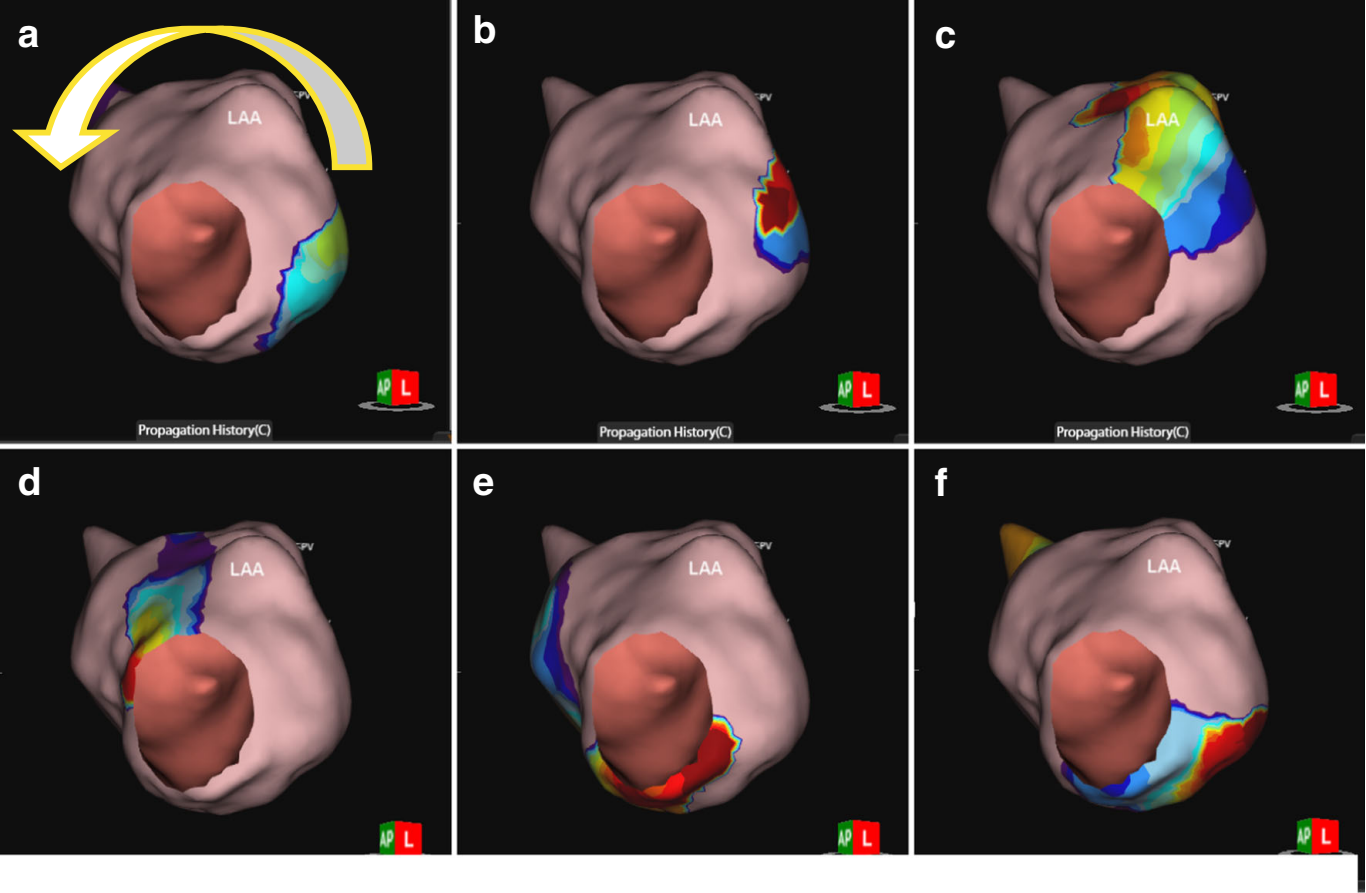
Mitral isthmus dependent atypical flutter: Panels (**a**), (**b**), (**c**), (**d**), (**e**), and (**f**) represent the electrical activation projected on the LA anatomy in different time steps throughout the full CL of the counter clock wise mitral isthmus dependent AFL. Yellow arrow is indicating the wave front. All panels show a LAO view of the LA

The remaining 4 patients (57%) exhibited reentrant tachycardias. In 3 patients the SuperMap algorithm detected mitral isthmus-dependent flutters (Fig. 4) with a tachycardia CL of 240, 270, and 420 ms, respectively. A mitral isthmus line was performed connecting the LIPV to the mitral annulus in a posterolateral position. In 1 patient additional epicardial applications were needed in the CS in order to terminate the tachycardia. Bidirectional block could be documented in all cases along the lines.

In 1 patient (16, 7%) the mapping revealed a macro-reentrant tachycardia with a CL 320 ms around a lateral RA atriotomy, which extended high towards the superior vena cava (SVC). The critical isthmus was located between crista terminalis and atriotomy. RF applications (30 W) were delivered on the lateral and posterolateral wall of RA converting to sinus rhythm and creating a line of block. Pharmacological stimulation failed to induce any arrhythmias in patients presenting re-entrant atrial tachycardias. The tachycardia characteristics and ablation target site are shown in Table 3.

**Table 3:** Tachycardia characteristics

Type of arrhythmia	Localization	CL (ms)	Ablation target site
AFL	LA	420	Mitral isthmus line
AT	LA	270	Anteroseptal RSPV
AFL	LA	240	Mitral isthmus line + epicardial CS
AT	LA	400	Posterior in proximity LIPV
AFL	RA	320	Critical isthmus between crista terminalis and atriostomy.
AFL	LA	270	Mitral isthmus line
AT	LA	235	Interatrial septum in proximity of fossa ovalis

## Complications

No minor or major complications occurred in the patient population.

## Discussion

To the best of our knowledge, this is the first study describing the feasibility, acute efficacy, and safety of the novel Acutus SuperMap Algorithm in the setting of post AF ablation ATs. The main findings of our first experience are (1) this novel algorithm could identify in all cases the arrhythmic substrate, (2) procedural times were by current standards low, and (3) no minor or major complications occurred.

Regular atrial tachycardias occur in a sizeable proportion of patients following AF ablation. The underlying mechanisms range from focal mechanisms to micro- or macro-reentrant tachycardias, often causing more symptoms to the patient than the initial AF [20]. Focal ATs can arise from anywhere in the atria but often arise from particular anatomical regions. Common locations in the RA include the tricuspid annulus, the crista terminalis, and the coronary sinus ostium. Para-Hisian ATs demonstrate properties consistent with tachycardias from other sites around the tricuspid annulus and are thus best considered to be a subset of annular ATs. In the LA, common sites include the pulmonary veins and the mitral annulus, particularly the left aortomitral continuity [20]. In patients, having undergone a PVI, focal mechanisms can play a role in reconnected veins causing regular atrial tachycardias. Macro-reentrant ATs often occur in patients with prior atrial ablation. The latter most commonly occur after linear ablation in the LA or RA. The incidence of AT after AF ablation varies, depending on the techniques and lesion sets employed to treat AF. Atrial tachycardias are less common after PVI if compared with patients treated with linear lesions and ablation of complex electrograms (5 vs 25%) [20]. While macro-reentrant arrhythmias are the most common form of AT following AF ablation, focal arrhythmias also occur in a minority of such patients. No matter what the nature of the AT, the underlying mechanism is paramount in guiding the ablation in the appropriate site [9]. The pivotal paper by Jais et al. [21] described the mapping and ablation in a series of atypical LA flutters. After a mean 1.4 procedures all arrhythmias could be successfully treated. However, given the initial experience with these complex arrhythmias, the procedures were time consuming with a mean of 339 ± 113 min per intervention. Patel et al. [22] reported a strategy for rapidly defining and eliminating the scar-related ATs typically encountered after ablation of atrial fibrillation. The mapping time using a PentaRay catheter was impressively low with a mean of 8 ± 3 min per procedure. However, the mean number of points per map was 365 ± 108, which might be considered too low by today’s standards in the high-density mapping era. Initial studies on mapping atypical flutters strongly relied on entrainment mapping. However, entrainment mapping has a number of limitations for mapping these arrhythmias. First, the measurement of the post-pacing interval might prove challenging and might either change or terminate the arrhythmia which is being mapped. In addition, it may be difficult to pace in areas of atrial scar which are part of the atrial flutter circuit. In some cases, the latter may only be accomplished with higher outputs that might distort local signals. A recent study by Winkle et al. [23] describing the advantage of ultra-high-density activation sequence mapping in the setting of atypical flutters showed that this method led to a successful termination of roughly 90% of arrhythmias in a cohort of 28 patients. The authors used a multipolar circular spiral mapping catheter (St Jude medical, USA) with an interelectrode distance of 1 mm. The mean number of collected points was 3815 ± 2576, and the mean mapping time from initiation of geometry to first RF delivery was 24 ± 11 min. The conclusion of this study is that it is possible using only high-density mapping, to map the entire arrhythmia circuit for most atypical atrial flutters and to successfully ablate them by targeting an area of slow conduction. The novel CD mapping system in conjunction with the SuperMap algorithm might offer considerable advantages with respect to the above-mentioned articles. First of all, considering this was a first experience with this new technology, mapping times were very low with a mean of 8.2 ± 1.0 min. This greatly contributed to short procedural times. Secondly, the amount of points gathered in a very short time was high with a mean of 5859.7 ± 4348.5 points per patients. This certainly offered very high-density mapping. However, in our opinion, the main advantage of this novel mapping system is that variations in cycle length and CS activation pattern changes do not influence the interpretation of the AT in question. In fact, the algorithm permits to map multiple clusters of arrhythmias and automatically distinguish the beats pertaining to the clinical arrhythmia. This is a strong limitation of sequential mapping with traditional 3D mapping systems, in which variation of cycle length or small changes in CS activation with the same CL inevitably hampers the accuracy of the map. Of note, in the study we performed entrainment mapping prior to ablation. Similar to the findings of Winkle et al., we believe that entrainment might not be needed as the SuperMap offers detailed information on the clinical arrhythmia clearly depicting the underlying mechanism. Finally, this procedure proved to be safe, and although our series of patients was small, no complications occurred. All our punctures were performed under ultrasound guidance, and specifically, no vascular adverse events in the site of puncture occurred despite the large 16 Fr sheath. Previous reports have shown excellent safety profile with this technique [24].

## Limitations

These study reports show the first experience with the SuperMap algorithm. This study was conducted in a small cohort of patients and is retrospective in nature. No definitive conclusions can be drawn and future larger studies are warranted to confirm our findings. We therefore believe that times for fluoroscopy will decrease significantly as our experience with this technology will increase.

## Conclusion

Our first experience with the novel noncontact CD map in conjunction with the SuperMap algorithm led to successful identification and ablation of the arrhythmic substrate in the setting of regular atrial tachycardias following index AF ablation.
