# Correction to: Novel noncontact charge density map in the setting of post-atrial fibrillation atrial tachycardias: first experience with the Acutus SuperMap Algorithm

**DOI:** 10.1007/s10840-020-00860-5

**Published:** 2020-09-12

**Authors:** Robbert Ramak, Gian-Battista Chierchia, Gaetano Paparella, Cinzia Monaco, Vincenzo Miraglia, Federico Cecchini, Antonio Bisignani, Joerelle Mojica, Maysam Al Housari, Dimitrios Sofianos, Shuichiro Kazawa, Ingrid Overeinder, Gezim Bala, Erwin Ströker, Juan Sieira, Thiago Guimaraes Osorio, Pedro Brugada, Carlo de Asmundis

**Affiliations:** Heart Rhythm Management Center, Postgraduate program in Cardiac Electrophysiology and Pacing, European Reference Networks Guard-Heart, Universitair Ziekenhuis Brussel, Vrije Universiteit Brussel, Laarbeeklaan 101, 1090 Brussels, Belgium

**Correction to: Journal of Interventional Cardiac Electrophysiology**

10.1007/s10840-020-00808-9

The article “Novel noncontact charge density map in the setting of post-atrial fibrillation atrial tachycardias: first experience with the Acutus SuperMap Algorithm”, written by Robbert Ramak, Gian-Battista Chierchia, Gaetano Paparella, Cinzia Monaco, Vincenzo Miraglia, Federico Cecchini, Antonio Bisignani, Joerelle Mojica, Maysam Al Housari, Dimitrios Sofianos, Shuichiro Kazawa, Ingrid Overeinder, Gezim Bala, Erwin Ströker, Juan Sieira, Thiago Guimaraes Osorio, Pedro Brugada, Carlo de Asmundis, was originally published electronically on the publisher’s internet portal on 08 July 2020 without open access. With the author(s)’ decision to opt for Open Choice the copyright of the article changed on 20 August 2020 to © The Author(s) 2020 and the article is forthwith distributed under a Creative Commons Attribution 4.0 International License, which permits use, sharing, adaptation, distribution and reproduction in any medium or format, as long as you give appropriate credit to the original author(s) and the source, provide a link to the Creative Commons license, and indicate if changes were made. The images or other third party material in this article are included in the article’s Creative Commons license, unless indicated otherwise in a credit line to the material. If material is not included in the article’s Creative Commons license and your intended use is not permitted by statutory regulation or exceeds the permitted use, you will need to obtain permission directly from the copyright holder. To view a copy of this license, visit http://creativecommons.org/licenses/by/4.0.

The original article has been corrected.

